# Prevalence, outcome and risk factors for postoperative pyothorax in 232 dogs undergoing thoracic surgery

**DOI:** 10.1111/jsap.12064

**Published:** 2013-04-15

**Authors:** L B Meakin, L K Salonen, S J Baines, D J Brockman, S P Gregory, Z J Halfacree, V J Lipscomb, K C Lee

**Affiliations:** Department of Veterinary Clinical Sciences, Royal Veterinary College, University of LondonHawkshead Lane, Hatfield, Hertfordshire, AL9 7TA; * School of Veterinary Sciences, University of BristolLangford, Bristol, BS40 5DU

## Abstract

**Objective:**

To determine the prevalence, outcome and risk factors for postoperative pyothorax in dogs undergoing thoracic surgery.

**Methods:**

Case records were reviewed retrospectively to identify dogs with post thoracic surgery pyothorax, defined as septic neutrophilic inflammation within the pleural space based on cytology and/or a positive bacterial culture of pleural fluid. Those identified were reviewed for potential risk factors for postoperative pyothorax based on biological plausibility and previously published data. These potential risk factors were explored by multivariable logistic regression.

**Results:**

Of 232 dogs undergoing thoracic surgery, 15 (6·5%) dogs developed pyothorax. Bacteria cultured included methicillin-resistant *Staphylococcus aureus* and multi-resistant *Escherichia coli*. Of these dogs, six died, four were euthanased and five were treated successfully. A diagnosis of idiopathic chylothorax [Odds Ratio (OR)=12·5, 95% Confidence Interval (CI)=2·7-58·5, P=0·001], preoperative intrathoracic biopsy (OR=14·3, 95% CI=1·7-118·7, P=0·014) and preoperative thoracocentesis (OR=11·2, 95% CI=1·6-78·2, P=0·015) were identified as independent risk factors for development of postoperative pyothorax.

**Clinical Significance:**

Idiopathic chylothorax, intrathoracic biopsy and prior thoracocentesis are independent risk factors for postoperative pyothorax, which was associated with a 67% mortality rate.

## Introduction

Surgical site infections (SSIs) remain an important concern in veterinary practice. This concern has increased recently following the identification of multi-antibiotic-resistant bacteria, primarily methicillin-resistant *Staphylococcus aureus* (MRSA) (Duquette & Nuttall 2004; Leonard & Markey 2008; Loeffler *et al*. 2005). SSIs have been defined as those occurring at the surgical site within 30 days of surgery or within 1 year of a surgery involving placement of a permanent implant (Weese 2008). They have been further classified into superficial infections involving the skin and subcutis, deep infections involving the underlying muscle and organ/space infections involving areas of the body deep to the surgical incision that were entered during the surgery. Overall, SSI rates of 5 to 6% have been reported, with lower rates of 2% for clean procedures and higher rates of up to 18% for contaminated or dirty procedures (Vasseur *et al*. 1988; Brown *et al*. 1997; Beal *et al*. 2000; Nicholson *et al*. 2002; Eugster *et al*. 2004).

Superficial and deep incisional SSIs should rarely be associated with mortality, though increased morbidity, hospitalization time and costs are incurred (Nelson 2011). Conversely organ/space SSIs including orthopedic prosthesis infection, pacemaker infections, septic peritonitis and pyothorax may result in significant morbidity and mortality (Girling & Innes 2006; Fine & Tobias 2007; Weese 2008; Grimes *et al*. 2011). To the authors’ knowledge, there is little data regarding the occurrence of postoperative pyothorax in dogs following thoracic surgery, yet this is a potentially life-threatening complication (Demetriou *et al*. 2002; Moores *et al*. 2007; Stewart & Padgett 2010).

The aims of this study were first to determine the prevalence of postoperative pyothorax in dogs undergoing thoracic surgery, secondly to report the treatment and outcome of these dogs and thirdly to identify risk factors associated with development of postoperative pyothorax.

## Materials And Methods

The study population comprised all dogs that underwent thoracic surgery between June 1998 and May 2007, identified by searching computerized hospital records and theatre logbooks. ‘Thoracic surgery’ included all thoracotomies irrespective of surgical approach and therefore included median sternotomies, intercostal thoracotomies and transdiaphragmatic approaches to the thorax, as well as thoracoscopic procedures. As the intention was to identify dogs which developed postoperative pyothorax due to a nosocomial infection, dogs were excluded from the study population if pyothorax was confirmed preoperatively, the preoperative diagnosis was strongly suggestive of bacterial contamination of the pleural space, e.g. external penetrating thoracic injury or oesophageal rupture or death occurred during or within 12 hours of surgery. Dogs were also excluded if their hospital record was incomplete. All surgeons were qualified or recognized specialists.

Postoperative pyothorax was defined as septic neutrophilic inflammation within the pleural space based on cytological evidence of bacteria within neutrophils and/or a positive bacterial culture of pleural fluid, within 30 days of surgery, in accordance with published definitions for SSI (Weese 2008). Patient details, method of diagnosis, treatment and outcome of postoperative pyothorax were recorded for all affected dogs.

A list of potential risk factors was compiled based on published risk factors for SSIs in the veterinary literature, their potential to promote bacterial contamination of the pleural space or to decrease the host immune response and the information which could be obtained reliably from the hospital records (Brown *et al*. 1997; Vasseur *et al*. 1988; Heldmann *et al*. 1999; Beal *et al*. 2000; Nicholson *et al*. 2002; Eugster *et al*. 2004; Weese 2008). These included patient factors (age, gender, weight, breed), preoperative factors (surgical disease or diagnosis, presence of pleural fluid detected by ultrasonography, prior thoracocentesis and/or pericardiocentesis, intrathoracic biopsy, placement of thoracostomy tubes, duration of hospitalization before surgery), surgical factors [National Research Council (NRC) surgical site contamination classification, approach to the thorax, anesthesia time, surgery time (measured from the first incision to completion of closure), use of perioperative antibiosis, number of thoracostomy tubes placed during surgery] and postoperative risk factors (need for repeat surgery due to complications, postoperative prophylactic antibiosis). Intrathoracic biopsy included biopsy of any internal structure, which did not require a surgical approach to the thorax, including fine needle aspirates and tru-cut biopsies, taken with ultrasound or CT guidance. Perioperative antibiosis was defined as the administration of antibiotic agents during anesthesia and up to 24 hours postoperatively.

Hospital protocol dictated pleural space drainage every 4 hours when thoracostomy tubes were placed. Drains were removed when fluid production was less than 2 mL/kg/day or had reached a steady low rate or following cessation of pleural air production in dogs with pneumothorax. If a dog was diagnosed with postoperative pyothorax while thoracic drains were still in place, the drains were maintained for therapeutic purposes. Therefore a diagnosis of postoperative pyothorax directly affected timing of drain removal. Consequently, whilst timing of thoracic drain removal was recognized as a potential risk factor for the development of postoperative pyothorax, it could not be assessed in the following analysis.

Associations between the development of postoperative pyothorax and potential risk factors were initially explored by univariate analysis using chi-squared, Fisher's exact, Mann–Whitney U or independent Student's *t*-tests as appropriate, with normalization of the data by logarithmic transformation where necessary. Factors associated with postoperative pyothorax in the univariate analysis (P≤0·20) were considered for inclusion in the multivariable logistic regression model using a stepwise approach. A factor was left in the model if the likelihood ratio test of comparing models with and without it showed evidence of a better fit with it (P<0·1). Each variable was also assessed for interaction with other variables using the likelihood ratio test method and, where significant interactions (P≤0·05) were detected, only one variable was included in the model at any one time. Ordered categorical variables were tested as linear and categorical effects. Model fit was evaluated by plotting the observed probabilities of postoperative pyothorax and those predicted by the model against the multiplicative effect of the included covariates. All statistics were performed using STATA9 (StataCorpLP). Results are presented as median (range) or mean (±sd) as appropriate.

## Results

### Study population

Two hundred and eighty-seven dogs underwent thoracic surgery during the study period. Fifty-five dogs were excluded because of preoperative pyothorax (41 dogs), death or euthanasia during or within 12 hours of surgery for reasons other than infection (10 dogs) and missing or incomplete case records (4 dogs).

Of the 232 included dogs, the median age was 48 (2 to 169) months. Fifty-four dogs were neutered males, 84 were entire males, 44 were neutered females and 50 were entire females. Sixty-two different breeds were represented with a median bodyweight of 22 (1 to 85) kg.

A summary of the primary diseases/diagnoses is presented in Table 1. Preoperatively, 48 dogs (20·7%) had pleural fluid, 60 (25·9%) had thoracocentesis performed, 24 (10·3%) had pericardiocentesis performed and 9 (3·9%) had an intrathoracic biopsy. Dogs were hospitalized for a median of 1 (0 to 21) day before surgery. No thoracostomy tubes were placed preoperatively. Antibiotics were not administered to any animal before the perioperative administration.

**Table 1:** Diagnosis leading to thoracic surgery in the 232 dogs included in the study

Diagnosis	Number
Patent ductus arteriosus	64
Primary pulmonary neoplasia	23
Idiopathic pericardial effusion	17
Idiopathic chylothorax	16
Spontaneous pneumothorax	14
Primary mediastinal neoplasia	9
Persistent right aortic arch	9
Tricuspid valve dysplasia	8
Cardiac neoplasia	7
Lung lobe torsion	7
Primary thoracic wall neoplasia	7
Diaphragmatic rupture	6
Mesothelioma	5
Secondary pulmonary neoplasia	5
Pulmonic stenosis	4
Restrictive pericarditis	3
Bronchial foreign body	3
Tetralogy of Fallot	2
Other	23

NRC surgical site contamination classification was clean for 163 (70·3%) and clean-contaminated for 69 (29·7%) surgeries. The thorax was approached by lateral thoracotomy in 155 (66·8%), median sternotomy in 64 (27·6%), thoracoscopy in 7 (3·0%), transdiaphragmatic in 4 (1·7%) and a combined transdiaphragmatic with median sternotomy in 2 (0·9%) dogs. Mean anesthesia time was 229 (±94) minutes and surgery time was 136 (±66) minutes). Perioperative antibiotics were administered to 195 dogs (84·1%). One or two thoracic drains were placed at the end of surgery in 218 (94·0%) and 11 (4·7%) dogs, respectively.

Within the study population, 5 (2·2%) dogs required a second surgery because of ongoing postoperative intrathoracic haemorrhage in each case. Antibiotics were continued beyond 24 hours after surgery in 15 (6·5%) dogs.

### Dogs with postoperative pyothorax

Fifteen (6·5%) dogs developed postoperative pyothorax. Median age of these dogs was 57 (14 to 114) months. The primary thoracic disease/diagnosis was idiopathic chylothorax in 9 (60·0%) dogs and spontaneous pneumothorax secondary to rupture of a pulmonary bleb, a pulmonary cyst, pulmonic stenosis, thoracic wall chondrosarcoma, mediastinal neoplasia and restrictive pericarditis in one (6·7%) dog each.

Other postoperative complications were identified in 9 (60·0%) of the 15 dogs that developed postoperative pyothorax. These were incisional wound infection and breakdown, hypoproteinaemia, systemic inflammatory response syndrome, laryngeal necrosis, urinary tract infection and liver disease with haemolytic anaemia.

Pyothorax was confirmed a median of 7 (4 to 24) days postoperatively. Bacterial cultures of pleural fluid were performed in 14 (93·3%) dogs with pyothorax and the diagnosis was based on cytology alone for the remaining dog. Bacteria isolated included MRSA (n=3, 20·0%), multi-antibiotic-resistant *Escherichia coli* (n=3, 20·0%), multi-antibiotic-resistant *Klebsiella spp*. (n=1, 6·7%), methicillin-resistant *Staphylococcus pseudintermedius* (n=1, 6·7%), methicillin-susceptible *S. aureus* and *S. pseudintermedius* (n=2 each, 13·3%), *Enterococcus faecalis* (n=4, 26·7%), *Streptococcus spp*. (n=3, 20·0%), *E. coli* (n=2, 13·3%), *Pasteurella spp*. (n=2, 13·3%) and *Pseudomonas aeruginosa* (n=1, 6·7%).

Treatment of pyothorax consisted of antibiotic administration and thoracic drainage in all dogs and thoracic lavage in 2 (13·3%) dogs. Antibiosis was initially broad-spectrum treatment which was changed if necessary once culture and susceptibility data were obtained. No dogs underwent a second surgical procedure to treat pyothorax. Of the dogs with postoperative pyothorax, 6 (40·0%) died a median of 9 (5 to 10) days following surgery, 4 (26·7%) were euthanased (three because of infection 10, 18 and 28 days postoperatively; the fourth was discharged on day 15 for euthanasia at home) and 5 (33·3%) were treated successfully and discharged a median of 14 days postoperatively (range 9 to 23). Of the dogs without postoperative pyothorax, 94 were confirmed to have survived because of their attendance at a 6-week postoperative consultation and 8 were confirmed to have died within the 30-day postoperative period. Dogs with postoperative pyothorax were more likely to die than those without (40·0 *versus* 7·8% mortality, P<0·001). Dogs that were infected with antibiotic-resistant bacteria were not significantly more likely to die than those infected with bacteria demonstrating an antibiotic susceptibility profile reported as normal for the species by a veterinary microbiologist (P=0·50) and culture of an antibiotic-resistant bacteria was not significantly associated with diagnosis (P=0·78).

### Risk factors for postoperative pyothorax: multivariable logistic regression analysis

Results of the univariate analysis are included in Table 2. All variables that demonstrated significance were positively associated with the development of pyothorax except for a diagnosis of non-neoplastic cardiovascular disease which was negatively associated. Using multivariable logistic regression, a diagnosis of idiopathic chylothorax [Odds Ratio (OR) 12·5, 95% Confidence Interval (CI) 2·7-58·5, P=0·001], preoperative intrathoracic biopsy (OR 14·3, 95% CI 1·7-118·7, P=0·014) and preoperative thoracocentesis (OR 11·2, 95% CI 1·6-78·2, P=0·015) were found to be significant independent risk factors (Table 3). Rise in surgical time (OR 1·3 for each 30-minute rise in surgery time, 95% CI 1·0-1·8, P=0·073) did not reach significance but remained in the final model as the model fit was better when it was included. No significant interactions between these four parameters were detected.

**Table 2:** Univariate analysis of potential risk factors for postoperative pyothorax

Variable	P-value
**Age**	**0·126**
**Gender**	**0·194**
**Bodyweight**	**0·002**
**Surgical disease – chylothorax**	**<0·001**
**Surgical disease – non-neoplastic cardiovascular**	**0·007**
**Preoperative pleural fluid**	**<0·001**
**Preoperative thoracocentesis**	**<0·001**
Preoperative pericardiocentesis	0·629
**Preoperative intrathoracic biopsy**	**0·050**
Preoperative duration of hospitalization	0·340
NRC surgical site contamination classification	0·790
**Surgical approach to the thorax**	**<0·001**
**Anesthesia time**	**0·001**
**Surgery time**	**<0·001**
Use of perioperative antibiosis	0·775
**Number of thoracic drains placed during surgery**	**<0·001**
Need for repeat surgery	0·213
Postoperative prophylactic antibiosis	0·958

Variables included in bold type were considered for inclusion in the multivariable logistic regression model

**Table 3:** Final multivariate logistic regression model for postoperative pyothorax

Variable	Odds Ratio	95% CI	P-value
Idiopathic chylothorax	12·5	2·7 to 58·5	0·001
Preoperative biopsy	14·3	1·7 to 118·7	0·014
Preoperative thoracocentesis	11·2	1·6 to 78·2	0·015
30-minute increase in surgery time	1·3	1·0 to 1·8	0·073

Given the high occurrence of postoperative pyothorax amongst the 16 dogs with idiopathic chylothorax, these cases were considered further. All dogs underwent median sternotomy for thoracic duct ligation, combined with ventral midline coeliotomy to facilitate lymphangiography. Nine dogs with postoperative pyothorax and six of seven (85·7%) dogs without postoperative pyothorax also underwent subtotal pericardectomy. Thoracic omentalization was performed in four of the nine (44·4%) dogs with and three of the seven (42·9%) dogs without postoperative pyothorax. One dog underwent lung cortication and this dog developed postoperative pyothorax. Surgery and anesthesia times for dogs undergoing surgery for chylothorax that did and did not develop postoperative pyothorax are shown in Table 4. No significant difference in anesthesia or surgery time was detected when comparing dogs with and without postoperative pyothorax (P=0·42 and P=0·48, respectively).

**Table 4:** Surgery and anesthesia times for dogs undergoing surgery for chylothorax

Variable	Postoperative Pyothorax	
	Yes	No
Anesthesia time (min)	319 ±13	296 ±23
Surgery time (min)	202 ±14	185 ±19

## Discussion

In this study, 6·5% of dogs undergoing thoracic surgery developed postoperative pyothorax and this complication resulted in death in 66·7% of the dogs. This infection rate falls within the previously reported rates of SSIs in small animal surgeries. Weese (2008) reported a median SSI rate of 4·5% with a range of 0·8 to 18·1%. However, previous reviews of the complications of thoracic surgery do not report a postoperative pyothorax rate of this magnitude (Bellenger *et al*. 1996; Burton & White 1996; Tattersall & Welsh 2006). The increased recognition of multi-antibiotic-resistant bacteria in veterinary patients may be responsible for this high rate of infection: these bacteria were implicated in almost half of dogs with postoperative pyothorax in this study. Equally the high rate of postoperative pyothorax may be because of pleural fluid culture and analysis being performed more frequently. Indeed review of previous reports of complications following thoracotomy suggests the possibility of SSIs in a significant percentage of dogs, but without confirmation by culture. For example, Tattersall and Welsh (2006) found wound complications in 31% between two and 14 days postoperatively, but with culture confirmation of infection in only 2% and Burton and White (1996) reported early wound infection in 3% and late sternal osteomyelitis, wound swelling and wound discharge in 6, 3 and 3% of cases, respectively. The mortality rate for postoperative pyothorax in this study was also higher than expected as previous studies reported mortality rates of up to 26% for spontaneous pyothorax (Demetriou *et al*. 2002; Rooney & Monnet 2002; Johnson & Martin 2007; Boothe *et al*. 2010). This could be because of concurrent underlying disease processes such as chylothorax combined with recent surgery compared to the young, healthy dogs which normally present with spontaneous pyothorax such as that secondary to a migrating foreign body.

In this study, idiopathic chylothorax was the risk factor most significantly associated with postoperative pyothorax in the multivariable analysis. This result was unexpected as multiple case series reporting surgical management of chylothorax do not site this as a significant complication (Birchard *et al*. 1988; Fossum *et al*. 2004; Hayashi *et al*. 2005; Carobbi *et al*. 2008; Allman *et al*. 2010; Bussadori *et al*. 2011). However, there are reports of pleural space infection in animals with chylothorax. Fossum *et al*. (1986) found bacterial cultures of pleural fluid were initially negative for 23 dogs with chylothorax, but following repeated thoracocentesis, pleural fluid cultures were positive in six. Pyothorax was also reported in a dog with severe bilateral fibrosing pleuritis, which had been managed non-surgically for 6 months (Fossum *et al*. 1992). These studies suggest that, whilst chyle has natural bacteriostatic properties, repeated thoracocentesis and chronic fibrosing pleuritis may increase the risk of pyothorax in animals with chylothorax.

Postoperative pleural infection associated with early mortality in animals managed surgically for chylothorax has also been reported in the veterinary literature in at least one study. Stewart and Padgett (2010) reported death in one of five dogs treated surgically for chylothorax, and postmortem examination confirmed acute fibrinopurulent pleuritis. It is interesting to note this study also used a combined median sternotomy and midline coeliotomy to approach the thorax and allow thoracic omentalization. Whether this approach or combination of procedures increases the risk of postoperative infection remains to be determined.

Identification of preoperative thoracocentesis and intrathoracic biopsy as risk factors may be due to direct inoculation of bacteria into the thoracic cavity during these procedures. Furthermore, these procedures are associated with clipping before induction of anesthesia for surgery, which has been reported to increase the risk of SSI threefold in veterinary patients (Brown *et al*. 1997).

Surgical time did not reach significance levels but showed a potential trend towards being positively associated with postoperative pyothorax in the multivariable model. This is unsurprising as surgery and/or anesthesia time have previously been shown to be significantly associated with SSI development (Vasseur *et al*. 1988; Brown *et al*. 1997; Heldmann *et al*. 1999; Beal *et al*. 2000; Nicholson *et al*. 2002; Eugster *et al*. 2004).

As this retrospective study was carried out at a veterinary referral centre, the limitations include inconsistent follow-up and possible under-reporting of postoperative pyothorax because of follow-up at local veterinary practices and death without postmortem examination. Furthermore, this study is from a single teaching hospital and may reflect differences in hospital-level factors. By increasing the number of animals, particularly dogs with postoperative pyothorax, the study power would be increased. In addition, because of the relatively high number of potential risk factors analysed (24) compared to the study sample size (232 dogs), some risk factors may have been found to be statistically significant by chance although this error was reduced by selection of potential risk factors which were biologically relevant.

In this study, postoperative pyothorax was associated with a high rate of mortality. A diagnosis of idiopathic chylothorax and preoperative thoracocentesis or intrathoracic biopsy were significant, independent risk factors contributing to the development of pyothorax in dogs following thoracic surgery. The association between chylothorax and postoperative pyothorax requires further study. Although additional studies are necessary to determine causality, it is suggested that decisions on whether to perform preoperative thoracocentesis or biopsy should consider the evidence presented in this study and be performed using strict aseptic technique.
